# Disease-free survival landmark analysis: a potential critical endpoint in triple-negative breast cancer

**DOI:** 10.1186/s40164-022-00308-8

**Published:** 2022-09-27

**Authors:** Fangchao Zheng, Tong Wei, Xue Wang, Feng Du, Jian Yue, Peng Yuan

**Affiliations:** 1 Department of VIP Medical Services, National Cancer Centre/National Clinical Research Center for Cancer/Cancer Hospital, Chinese Academy of Medical Sciences and Peking Union Medical College, Beijing, 100021 China; 2 Department of medical oncology, Shandong Cancer Hospital and Institute, Shandong First Medical University and Shandong Academy of Medical Sciences, 250117 Jinan, China; 3 Key Laboratory of Carcinogenesis and Translational Research (Ministry of Education/Beijing), The VIPII Gastrointestinal Cancer Division of Medical Department, Peking University Cancer Hospital and Institute, Beijing, 100021 China

**Keywords:** Landmark analysis, Platinum-based, Anthracycline-based, Triple-negative breast cancer, Prognosis

## Abstract

Taxanes plus carboplatin (TP) regimen may be an acceptable alternative adjuvant chemotherapy strategy in patients with triple-negative breast cancer (TNBC); however, the difference with the anthracycline-based regimen is yet to be clarified. Therefore, this study aimed to assess the difference between platinum-based and anthracycline-based regimens in prolonging the survival time in TNBC. Using exploratory landmark analysis, we found that the platinum-based TP regimen offers a longer disease-free survival (DFS) than the anthracycline-based regimen in TNBC patients with a DFS of > 4 years.

To the Editor,

Breast cancer, as the most common cancer in females, threatens women’s health worldwide [1]. Triple-negative breast cancer (TNBC) is a solid malignancy with negative expression of estrogen receptor (ER), progesterone receptor (PR), and human epidermal growth factor receptor-2 (HER2), which accounts for 15%-20% of breast cancer [2]. The anthracycline-based regimen, such as epirubicin plus cyclophosphamide followed by docetaxel or paclitaxel (ECT) regimen, is considered a standard adjuvant chemotherapy regimen and improves survival outcomes of early TNBC [3, 4]. In our previous study, [5] adjuvant carboplatin plus docetaxel or paclitaxel (TP) showed non-inferiority for disease-free survival (DFS) and overall survival (OS) compared with ECT regimen in TNBC patients.

The platinum-based regimen is an effective alternative adjuvant chemotherapy regimen and is widely used in neoadjuvant chemotherapy for increased pCR rate in patients with TNBC, [4, 6] but it is still unclear whether a platinum-based regimen as adjuvant treatment in TNBC patients has a difference of survival benefit compared with an anthracycline-based regimen. Landmark analysis based on the DFS and OS time can minimize the immortal time bias induced by including events in the hazard model, [7, 8] and provide potential evidence of this difference. Thus, we excavated the landmark analysis aiming to investigate the role of platinum-based adjuvant settings in TNBC patients (ClinicalTrials.gov identifier NCT01150513). The final date of follow-up was January 20, 2021, with a median follow-up of 97.6 months. The Kaplan–Meier method and Breslow test were used to evaluate the prognostic value of early TNBC patients with 2-sided tests set at *P* < 0.05. The landmark analysis was performed using EmpowerStats software (version EmpowerR 2.2, X&Y Solutions, USA).

Based on our previous study, [5] we enrolled all TNBC patients treated with the TP regimen or the ECT regimen in the landmark analysis at 4 years. For TNBC patients with a DFS of > 4 years, a total of 125 (81.2%) patients in the ECT regimen and 127 (82.5%) patients in the TP regimen were analyzed in the study. TNBC patients (a DFS of > 4 years) were well balanced between the ECT regimen and TP regimen, and the detailed clinicopathological characteristics were seen in Table 1. As shown in Fig. 1, in TNBC patients with a DFS of ≤ 4 years, DFS (HR, 1.59; 95% CI, 0.86–2.95; *P* = 0.32; Fig. 1A) and OS (HR, 1.24; 95% CI, 0.61–2.54; *P* = 0.26; Fig. 1B) had no difference between the TP regimen and the ECT regimen. In TNBC patients with a DFS of > 4 years, the TP regimen had a longer DFS (HR, 0.28; 95% CI, 0.10–0.74; *P* = 0.01) (Fig. 1A) and was not associated with a better OS (HR, 0.39; 95% CI, 0.05–2.75; *P* = 0.4) (Fig. 1B) than the ECT regimen.

**Table 1:** Clinicopathological features in triple-negative breast cancer patients with a DFS > 4 years (%)

	ECT arm (n = 125)	TP arm (n = 127)	*P* value
Ages (median [IQR])	48 (42.0—53.0)	48 (42.5—57.0)	0.49
Menopasusal status			0.98
Premenopausal	75 (60.00)	75 (59.06)	
Postmenopausal	50 (40.00)	52 (40.94)	
Histological type			0.20
Ductal	117 (93.60)	117 (92.13)	
Lobular	4 (3.20)	1 (0.79)	
Medullary	3 (2.40)	4 (3.15)	
Others	1 (0.80)	5 (3.94)	
Histological grade			0.30
Grade 2	35 (28.00)	25 (19.69)	
Grade 3	78 (62.40)	88 (69.29)	
Missing or unkown	12 (9.60)	14 (11.02)	
Ki67			0.54
< 20%	15 (12.00)	13 (10.24)	
20% ≤ to < 50%	41 (32.80)	43 (33.86)	
≥ 50%	65 (52.00)	70 (55.12)	
Missing or unkown	4 (3.20)	1 (0.79)	
pT			0.35
pT1	67 (53.60)	67 (52.76)	
pT2	56 (44.80)	60 (47.24)	
pT3	2 (1.60)	0 (0.00)	
pN			0.42
pN0	81 (64.80)	93 (73.23)	
pN1	35 (28.00)	29 (22.83)	
pN2	4 (3.20)	3 (2.36)	
pN3	5 (4.00)	2 (1.57)	
pTNM stage			0.53
stage 1	47 (37.60)	50 (39.37)	
stage 2	69 (55.20)	72 (56.69)	
stage 3	9 (7.20)	5 (3.94)	
Intravascular invasion (%)			0.27
Yes	99 (79.20)	110 (86.61)	
No	17 (13.60)	10 (7.87)	
Missing or unkown	9 (7.20)	7 (5.51)	
PD-L1 status			0.16
negative	69 (55.20)	57 (44.88)	
postive	24 (19.20)	24 (18.90)	
Missing or unkown	32 (25.60)	46 (36.22)	
Surgery			0.07
Radical surgery	94 (75.20)	81 (63.78)	
Breast conserving	31 (24.80)	46 (36.22)	

*DFS* disease-free survival, *ECT*, docetaxel or paclitaxel followed by epirubicin plus cyclophosphamide, *TP* docetaxel or paclitaxel plus carboplatin

**Fig. 1:**
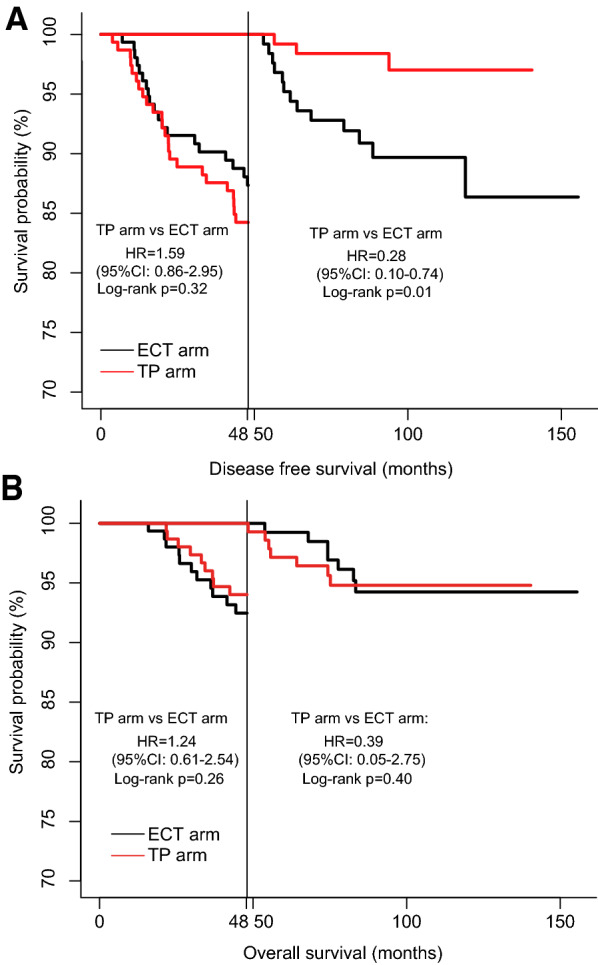
Landmark analysis plots showing the DFS and OS rates of different subgroups. (A) DFS of the subgroup with DFS of ≤ 4 years and DFS of > 4 years. (B) OS of the subgroup with a DFS of ≤ 4 years and DFS of > 4 years. TP, docetaxel or paclitaxel plus carboplatin; ECT, epirubicin and cyclophosphamide followed by docetaxel or paclitaxel; DFS, disease-free survival; OS, overall survival

This is the first landmark analysis assessing the difference in therapeutic effect between a platinum-based regimen and an anthracycline-based regimen as adjuvant treatment in TNBC patients, which showed that the TP regimen seems to have a longer DFS than the ECT regimen with a life expectancy of more than 4 years. The following reason may explain the differences. Firstly, the 5-year DFS rate of the TP regimen in our previous study (84.4%) was almost consistent with that in another study (86.5%) [4]. However, the results in our control group were inconsistent with that in the other study, probably because the ECT regimen administered in our control group was stronger than that of cyclophosphamide, epirubicin plus fluorouracil followed by the docetaxel regimen used in the previous study. Secondly, a recent study showed that high-dose anthracycline-based chemotherapy elicits a state of immunological dormancy and promotes resistance to chemotherapy in ER-negative BC patients (including those with TNBC) receiving adjuvant chemotherapy [9]. Based on the results of a previous study, anthracycline-based ECT regimen may evade chemotherapy by going senescence, leading to TNBC relapsed. Nevertheless, the TP regimen may be a potentially preferred adjuvant chemotherapy regimen for TNBC patients, especially, in whom, for some reason, the standard anthracycline-taxane regimen is not being used.

## Data Availability

The datasets used and/or analysed during the current study are available from the corresponding author on reasonable request.

## Abbreviations

TNBC: Triple-negative breast cancer
ER: Estrogen receptor
PR: Progesterone receptor
HER2: Human epidermal growth factor receptor-2
ECT: Epirubicin plus cyclophosphamide followed by docetaxel or paclitaxel regimen
TP: Carboplatin plus docetaxel or paclitaxel
DFS: Disease-free survival
OS: Overall survival
